# The association of religious affiliation with cholesterol levels among South Asians: the Mediators of Atherosclerosis in South Asians Living in America study

**DOI:** 10.1186/s12872-019-1045-z

**Published:** 2019-03-29

**Authors:** Grishma Hirode, Eric Vittinghoff, Nazleen H. Bharmal, Namratha R. Kandula, Alka M. Kanaya

**Affiliations:** 1 OakCare Medical Group, Highland Hospital, 1411 E 31st St, Oakland, CA 94602 USA; 2 UCSF Department of Epidemiology and Biostatistics, 550 16th St, San Francisco, CA 94158 USA; 3 U.S. Department of Health and Human Services, 200 Independence Avenue, Washington DC, 20201 USA; 4 Department of General Internal Medicine, Northwestern University Feinberg School of Medicine, 750 N Lake Shore Drive, 6th Floor, Chicago, IL 60611 USA; 5 UCSF Division of General Internal Medicine, 1545 Divisadero Street, Suite 311, San Francisco, CA 94115 USA

**Keywords:** South Asian, Religion, Cholesterol, Lifestyle, Lipids, Lipoproteins, Religious affiliation

## Abstract

**Background:**

South Asians have disproportionately high rates of cardiovascular disease. Dyslipidemia, a contributing factor, may be influenced by lifestyle, which can vary by religious beliefs. Little is known about South Asian religions and associations with dyslipidemia.

**Methods:**

Cross-sectional analyses of the MASALA study (*n* = 889). We examined the associations between religious affiliation and cholesterol levels using multivariate linear regression models. We determined whether smoking, alcohol use, physical activity, and dietary pattern mediated these associations.

**Results:**

Mean LDL was 112 ± 32 mg/dL, median HDL was 48 mg/dL (IQR:40–57), and median triglycerides was 118 mg/dL (IQR:88–157). Muslims had higher LDL and triglycerides, and lower HDL, while participants with no religious affiliation had lower LDL and higher HDL. The difference in HDL between Muslims and those with no religious affiliation was partly explained by alcohol consumption.

**Conclusions:**

Religion-specific tailoring of interventions designed to promote healthy lifestyle to reduce cholesterol among South Asians may be useful.

## Background

Dyslipidemia is a significant risk factor for cardiovascular disease (CVD), and is a major public health challenge [1–3]. Dyslipidemia is characterized by higher concentrations of total cholesterol, LDL-cholesterol and triglycerides, and lower HDL-cholesterol [4, 5]. Compared to other racial/ethnic groups, South Asians (individuals from India, Pakistan, Bangladesh, Nepal, and Sri Lanka) have higher rates of dyslipidemia-related conditions, such as atherosclerosis and coronary heart disease (CHD) [6–13]. South Asians have become one of the fastest growing immigrant groups in the U.S., with nearly 5.1 million South Asians in the U.S. currently [1, 14].

While certain individuals are genetically predisposed to dyslipidemia-related conditions, the high prevalence of dyslipidemia among South Asians may also be driven by acculturation, socio-economic status, and lifestyle behaviors such as high fat and calorie dense diets and physical inactivity [4, 5, 15–18]. Cultural and religious norms often influence lifestyle behavior choices [15, 19–23]. In a prior study, religious affiliation was positively associated with healthy lifestyle choices [24]. For example, Seventh-day Adventists (SDA) have traditionally had lower rates of chronic diseases, including obesity, CVD and diabetes than the general population because of their prescriptive lifestyle [25]. Along with adequate exercise and rest, Adventists are encouraged to adopt a healthful lifestyle including a vegetarian diet, avoiding caffeine-containing beverages, rich and highly refined foods, hot condiments and spices, and abstaining from alcohol, tobacco and narcotics [25, 26]. A 30-day diet and lifestyle intervention (physical activity daily, stress management techniques, sleep, and life balance) showed greater improvement in blood lipids and other health outcomes in non-SDA compared to SDA because the SDA had healthier behaviors at baseline [25].

Among religions practiced in South Asia, Islam prohibits alcohol due to the risk of alcohol-related diseases, and pork ingestion [27, 28]. Muslims believe that CVD is significantly decreased through these lifestyle promotions [29, 30]. Hinduism promotes a *sattvic* diet that is meant to include food and eating habits that are “pure, essential, natural, vital, energy-containing, clean, conscious, true, honest, wise” [15, 31, 32] such as seasonal foods, fruits, dairy products, nuts, seeds, oils, ripe vegetables, legumes, whole grains, and non-animal based proteins [33]. Sikhism prohibits tobacco use, discourages alcohol use, and promotes yoga and meditation [34].

Religion has already been proposed and used as a means of enhancing patient and community awareness of diabetes [34]. Incorporation of religion and culture-specific motivational and therapeutic strategies improves patient–physician communication and bonding, facilitates appropriate patient-centered care, and provides a framework upon which desired outcomes can be achieved [35–38]. Behavioral interventions targeting religion-specific modifiable factors for dyslipidemia—namely diet and physical activity—hold most promise for addressing these disproportionate disease rates among South Asians [15, 39, 40]. Using data from the Mediators in Atherosclerosis in South Asians Living in America (MASALA) study, we examined the association between religious affiliation and cholesterol levels among a religiously heterogeneous South Asian immigrant cohort. We also determined whether lifestyle behaviors such as smoking, alcohol intake, the amount of physical activity and dietary patterns would explain these associations. We hypothesized that different religious affiliations within the heterogeneous South Asian community may affect dietary practices and physical activity differentially due to religion-specific lifestyle prescriptions/proscriptions which would be associated cholesterol levels [41–44].

## Methods

### Data source

We performed a cross-sectional analysis of the baseline data from the MASALA study. The MASALA study is a community-based prospective cohort of 906 South Asians, free of CVD at baseline, enrolled from two geographical regions in the U.S. between October 2010 and March 2013 [6]. The University of California, San Francisco (UCSF) and Northwestern University (NWU) institutional review boards approved the protocol. Missing and refused observations were dropped from our multivariable analyses and thus the total sample size for this analysis was 889. There were 3 participants who had missing values for all cholesterol outcome variables. Another 7 participants were missing calculated LDL values, and an additional 14 were missing dietary pattern data. Measurements obtained at baseline included (but not limited to) socio-demographic information, lifestyle factors, anthropometric measurements, and fasting blood samples for biochemical risk factors [42].

### Cholesterol levels

After a requested 12-h fast, phlebotomy was conducted by certified phlebotomists or nurses to determine lipoprotein and lipid levels in milligrams per deciliter [6]. Total cholesterol, HDL and triglycerides were measured by enzymatic methods (Quest, San Jose, CA) and plasma LDL was consequently estimated using the Friedewald formula [45].

### Religious affiliation

Participants self-reported their religious affiliation with response items that included Hinduism, Sikhism, Islam, Jainism, Christianity, Buddhism, Zoroastrianism, or none. We collapsed these religious affiliations into fewer categories. We created a category combining those who self-reported Hinduism or Jainism because they have similar religious dietary proscriptions. Due to small numbers we classified participants who reported Christianity, Buddhism, Zoroastrianism, and those who reported more than one affiliation as “Other”.

### Covariates/mediators

We included socio-demographic characteristics and cholesterol medication use as covariates, and smoking, alcohol intake, the amount of physical activity and dietary patterns as mediators in our analysis. Socio-demographic characteristics included age, sex, years of life spent in the U.S., overall health status, marital status, educational attainment, health care access, insurance status and family income. Cholesterol medication use was assessed by medication inventory and included statin, fibrate, niacin, ezetimibe, or colesevelam use.

Smoking status was assessed based on whether the participant had never smoked, was a former smoker or currently smoked cigarettes. We categorized alcohol intake based on the number of alcoholic beverages consumed per week. Physical activity was assessed using the Typical Week’s Physical Activity Questionnaire [46] and was calculated as Metabolic Equivalent of Task (MET)-minutes of physical activity per week. Diet was assessed using the Study of Health Assessment and Risk in Ethnic Groups (SHARE) Food Frequency Questionnaire, which was developed and validated in South Asians in Canada, and included items unique to the South Asian diet [42, 47, 48]. Principal component analysis identified three distinctive dietary patterns; each respondent was assigned to the pattern for which he/she had the highest factor score [49]. The three major dietary patterns observed were Western (alcohol, coffee, eggs, fish, pasta, pizza, poultry, red meat, refined grains, vegetable oil; negative for whole grain, low-fat dairy, legumes), Sweets and Refined Grains (positive for added fat, butter/ghee, fried snacks, fruit-juice, high-fat dairy, sugar-sweetened beverages, legumes, potatoes, refined grains, rice, snacks, sweets, whole grains; negative for vegetable oil and nuts), and Fruits and Vegetables (positive for fruit, fruit juice, legumes, low-fat dairy, vegetable oil, nuts, vegetables, whole grains) [49].

### Statistical analysis

We described the prevalence of baseline characteristics in the study population. Between-group comparisons utilized chi-squared testing. Multivariable linear regression analysis was used to estimate the independent associations of religious affiliation with LDL, HDL and triglyceride levels with the base models adjusted for age, sex and cholesterol medication use. Variables with biological plausibility were included in the multivariate model. Then, to examine potential mediation of the effects of religious affiliation by lifestyle behaviors, we added smoking status, alcohol intake, physical activity and dietary pattern to the base model. Finally, we tested for modification of the effects of religious affiliation by sex and none were found. A *p*-value of less than 0.05 indicated statistical significance.

In fitting the linear models, we log-transformed LDL, HDL and triglyceride levels to meet normality assumptions, so that exponentiated coefficient estimates are interpretable as percentage between-group differences in average cholesterol levels. We calculated and presented adjusted means before and after the addition of mediators, using standardization. We performed pair-wise comparisons to analyze differences in cholesterol levels between groups.

The analysis was conducted using STATA/SE Version 14.0 (StataCorp, College Station, TX).

## Results

**Fig. 1:**
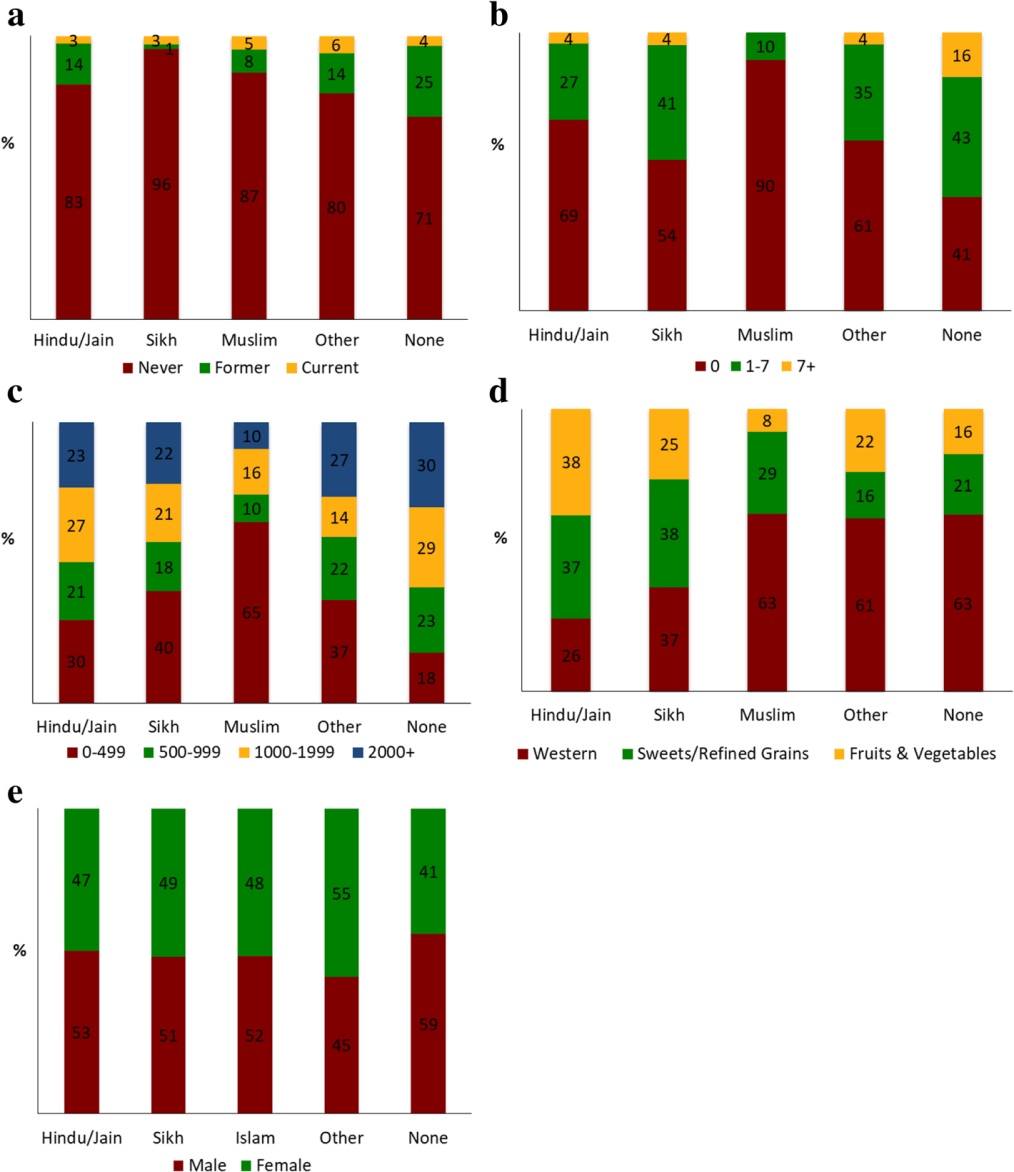
Distribution by lifestyle behaviors and sex based on religious affiliation: **a** smoking status (*p* = 0.014), **b** alcohol intake [drinks/week] (*p* < 0.001), **c** physical activity [MET-min/week] (*p* < 0.001), **d** dietary pattern (*p* < 0.001), and **e** sex (*p* = 0.69)

Of the 889 South Asians in this analysis, the average age was 55 ± 9 years, 53% were men, and 29% were taking cholesterol medications (Table 1). Cholesterol medication use did not differ significantly by religious affiliation (*p* = 0.36). On average, half of participants’ lives were spent in the U.S. The majority of participants identified with Hinduism/Jainism (74%), followed by Sikhism (8%), Islam (7%), other religious affiliations (6%), and no religious affiliations (6%). A majority of the participants had never smoked (83%) and did not consume alcohol on a weekly basis (67%). Smoking status, alcohol intake, physical activity, and dietary pattern differed significantly by religious affiliation, but there were no differences by sex (Fig. 1). The overall mean LDL was 112 ± 32 mg/dL, median HDL was 48 mg/dL (IQR: 40–57), and median triglycerides was 118 mg/dL (IQR: 88–157).

**Table 1:** Characteristics of MASALA study participants, 2010-2013^a^

Characteristic	*n*	% or mean (SD)
Age (years)	889	55.3 (9.4)
Gender (%)		
Male	471	53.0
Cholesterol Medication Use (%)		
Yes	259	29.1
Religious affiliation (%)		
Hinduism/Jainism	654	73.6
Sikhism	68	7.7
Islam	62	7.0
Other	49	5.5
None	56	6.3
Smoking status (%)		
Never	740	83.2
Former	121	13.6
Current	28	3.2
Alcohol Intake (drinks/week) (%)		
*0*	596	67.0
*1–7*	253	28.5
7+	40	4.5
Physical Activity (MET-min/week) (%)		
0–499	288	32.4
500–999	177	19.9
1000–1999	221	24.9
2000+	203	22.8
Dietary Pattern (%)		
Western	297	33.3
Sweet/Refined Grains	304	34.2
Fruits & Vegetables	288	32.4
*LDL (mg/dL)*	882	111.5 (32.0)
*HDL (mg/dL)*	889	50.1 (13.3)
*Triglycerides (mg/dL)*	889	131.4 (71.5)

^a^ Totals may not sum to 100% due to rounding error

In the base model, Islamic religious affiliation (or those of Muslim faith) was associated with higher LDL, while no religious affiliation was associated with lower LDL (Table 2). In particular, Muslims had 9% (95% CI: 2 to 17%, *p* = 0.02) higher LDL compared to Hindus/Jains. Muslims also had lower HDL, while those with no religious affiliation had higher HDL. In particular, HDL was lower by 7% (95% CI: -12 to − 0.3%, *p* = 0.04) among Hindus/Jains, and by 9% (95% CI: -17 to − 1%, *p* = 0.03) among Muslims compared those with no religious affiliation. Muslims also had higher triglyceride levels; triglycerides were 20% (95% CI: 2 to 42%, *p* = 0.03) higher among Muslims compared to those with other religious affiliations, and 20% (95% CI: 3 to 41%, *p* = 0.02) higher compared to those with no religious affiliation.

**Table 2:** Adjusted Means by Religious Affiliation for Cholesterol Levels in Serially Adjusted Models

	LDL		HDL		Triglycerides	
	Base Model^a^ (*n* = 882)	Fully Adjusted Model^b^ (*n* = 882)	Base Model^a^ (*n* = 889)	Fully Adjusted Model^b^ (*n* = 889)	Base Model^a^ (*n* = 889)	Fully Adjusted Model^b^ (*n* = 889)
	Mean (95% CI)	Mean (95% CI)	Mean (95% CI)	Mean (95% CI)	Mean (95% CI)	Mean (95% CI)
Religious Affiliation						
Hinduism/ Jainism	105.8 (103.6, 108.0)^*^	106.0 (103.8, 108.2)^†^	48.2 (47.3, 49.1)	48.2 (47.4, 49.1)	118.3 (114.3, 122.3)	118.1 (114.2, 122.2)
Sikhism	111.3 (104.3, 118.7)	112.0 (104.9, 119.5)	50.4 (47.7, 53.4)	50.1 (47.4, 53.0)	118.5 (106.7, 131.7)	119.5 (107.4, 132.9)
Islam	115.4 (107.8, 123.5)	114.0 (106.2, 122.4)	46.8 (44.1, 49.6)	48.2 (45.4, 51.2)	131.8 (118.1, 147.2)	127.7 (113.8, 143.3)
Other	106.4 (98.6, 114.7)	105.8 (98.0, 114.2)	47.5 (44.5, 50.8)	47.3 (44.3, 50.5)	109.7 (97.0, 124.2)^§^	110.3 (97.3, 124.9)
None	105.2 (98.1, 112.9)	104.0 (96.7, 111.8)	51.5 (48.5, 54.8)^‡^	50.1 (47.1, 53.4)	109.7 (97.7, 123.1)^**^	113.6 (100.9, 127.8)

^a^Adjusted for age, sex, and cholesterol medication use

^b^Fully adjusted model with smoking status, alcohol intake, physical activity and dietary pattern added to the base model

^*^p = 0.02 for Hinduism/Jainism compared to Islam

^†^*p* = 0.05 for Hinduism/Jainism compared to Islam

^‡^*p* = 0.03 for None compared to Islam; *p* = 0.04 for None compared to Hinduism/Jainism

^§^*p* = 0.03 for Other compared to Islam

^**^p = 0.02 for None compared to Islam

Adjusting for all of the mediators concurrently for each outcome (LDL, HDL or triglycerides) had minimal effects on the adjusted means for each group (Table 2). Only two measures of mediation were nominally statistically significant: specifically, 60% (95% CI: 1 to 118%) of the difference in adjusted mean HDL between Muslims and those with no religious affiliation was explained by lifestyle behaviors, and in particular, alcohol intake explained 49% (95% CI: 0.4 to 97%).

## Discussion

In a cross-sectional analysis of a community-based cohort of South Asians without known CVD, Muslims had higher LDL and triglyceride levels, and lower HDL levels, while participants with no religious affiliation had lower LDL levels and higher HDL levels. Hindus/Jains also had lower HDL levels compared to those with no religious affiliation. However, lifestyle behaviors did little to explain the associations between religious affiliation and cholesterol levels. The sole exception was the difference in HDL levels between Muslims and those with no religious affiliation, which was partly explained by lifestyle behaviors, in particular, by alcohol consumption.

While still controversial, reference standards for lipoprotein and lipid levels differ for South Asians compared to other ethnicities due to their markedly elevated risk for heart disease. For a person of average risk, an LDL level of ≤100 mg/dL, HDL level of ≥50 mg/dL for women and ≥ 40 mg/dL for men, and a triglyceride level of < 150 mg/dL [50, 51] are considered optimal. Age-, sex- and cholesterol medication use-adjusted mean LDL was slightly higher than the optimal range for all religious affiliations, the adjusted mean HDL was borderline, and the adjusted mean triglycerides was well below the optimal range in all groups. Thus, this study had a relatively healthy cohort with no major differences by religious affiliation.

While there is a paucity of studies on the relationship between religious affiliation and cholesterol levels in South Asian immigrants, our results are consistent with a previous study of South Asians in the U.K. that found lower HDL and higher triglycerides among Muslims compared to individuals of other religious affiliations [11]. Muslims are less likely to drink alcohol, and in our sample, majority of the Muslims did not consume alcohol. We found that moderate alcohol intake was associated with better lipid outcomes, specifically, higher HDL and lower triglyceride levels, independent of religious affiliation. Intervention studies in human subjects have shown ethanol-mediated HDL elevation, generally considered an important contributor to the beneficial and cardioprotective effects of moderate drinking [52–54]. However, socioeconomic status, physical activity, and diet also play a role [55]. Effects of alcohol consumption, moderate or otherwise, should be viewed as part of broader social, cultural, and lifestyle issues rather than in isolation [55, 56]. Cross-cultural prospective studies have shown that simply correlating the amount of alcohol consumed with CVD outcomes is inadequate as outcomes relate strongly to the patterns of drinking [55–57].

We examined physical activity and diet as other lifestyle behaviors that are influenced by religion. In general, South Asians have reportedly low rates of physical activity, and in our sample, one-third of participants were relatively sedentary (< 500 MET-min/week) [8, 58, 59]. Cultural, religious and gender norms have also been shown to be a barrier to physical activity among South Asian women [60]. Considerable evidence supports that physical activity and exercise have a positive impact on abnormal lipids [61–71]. In our sample, Muslims had the most sedentary lifestyle while those with no religious affiliations had the most active lifestyle. Hindus/Jains were more active compared to Muslims. This could be a factor for the unfavorable cholesterol outcomes among Muslims compared to those with no religious affiliation, and may explain the higher LDL levels among Muslims compared to Hindus/Jains.

In addition to the sedentary lifestyle and innate genetic predisposition, the consumption of sugar-sweetened beverages and added sweeteners have been proven to specifically lower HDL [49, 58, 72], and diets rich in fruits and vegetables, low-fat dairy products, reduced saturated fat, total fat and cholesterol content have been proven to substantially lower the total and LDL-cholesterol concentration [73, 74]. In our sample, Hindus/Jains were more likely to consume fruits and vegetables diet but also sweets and refined grains. Thus, the beneficial effects of a healthy diet may have been masked resulting in lower HDL levels compared to those with no religious affiliation. Muslims were least likely to eat fruits and vegetables, thereby possibly contributing to some of the higher LDL and lower HDL levels among Muslims.

While smoking is considered an important conventional risk factor, a previous study found that the levels of smoking did not account for the high rates of CHD in South Asians, and measures to reduce the habit would only have a modest impact on total mortality in South Asians [75, 76]. Smoking did not mediate the association between religious affiliation and cholesterol levels in our current study, possibly due to the low rates of current smokers in this sample.

This study has limitations. Given the cross-sectional design, we are unable to draw causal inferences about the association between religious affiliation and cholesterol levels. Measures of religiosity, such as adherence to religious tenets or practices, were not measured, and may provide further explanation for the positive association; future questionnaires will include more detailed questions on religious participation. Dietary patterns may not have adequately captured the nuances between some religious groups [49]. For example, Muslims are more likely to eat meat and refrain from alcohol [49, 77] but majority of the Muslims were placed in the animal protein dietary pattern group, which included alcohol. The category “other” for religious affiliation may contain religions with contradictory dietary proscriptions. There may have been some loss of statistical power due to use of categorical predictors. While representative of the South Asian community in the U.S., majority of the sample was well educated, which may limit capturing the effect of socioeconomic status, and majority of the participants affiliated with Hinduism/Jainism, which resulted in unequal group sizes thereby causing further loss of statistical power [6]. Inflation of some of the fully adjusted coefficients relative to the base model suggests negative confounding. Bias may also arise from uncontrolled confounding of the association between the mediators and the outcomes.

The religious and cultural community are often synonymous for certain South Asian groups, and the social influences within these groups may encourage or discourage behaviors that influence CVD risk [17, 20, 78–85]. Specifically in the U.S. where South Asians comprise 1.5% of the total population, we speculate that understanding different religious proscriptions would be clinically important for patient care. CVD outcomes are strongly associated with alcohol intake patterns, and drinking patterns are a result of the underlying social atmosphere. Although moderate drinking has been associated with better CVD outcomes, alcohol is prohibited for Muslims, and thus alternate modifications may be necessary. Based on our study, Muslims were least likely to eat fruits and vegetables, while Hindus ate greater amounts of sweets and refined grains. Thus, knowing this information with respect to diet and nutrition among different religious groups can help to tailor targeted interventions for patients.

## Conclusions

In conclusion, our results suggest that religion is associated with cholesterol levels in South Asians, and that some lifestyle behaviors may partially explain this risk. Specifically, the lack of alcohol consumption may contribute to lower HDL-cholesterol levels among Muslims. Behavioral lifestyle interventions to improve lipoprotein and lipid levels may be more effective if they take into account the influence of religious affiliation on lifestyle among the diverse South Asian population. Future interventional studies are required to confirm this association between South Asian religious groups, lifestyle behaviors and CVD risk.

## Abbreviations

CHD: Coronary Heart Disease
CI: Confidence Interval
CVD: Cardiovascular Disease
HDL: High-density Lipoprotein
IRB: Institutional Review Board
LDL: Low-density Lipoprotein
MASALA: Mediators in Atherosclerosis in South Asians Living in America
MESA: Multi-Ethnic Study of Atherosclerosis
MET: Metabolic Equivalent of Task
PTE: Proportion of total effect
SDA: Seventh-day Adventists
SHARE: Study of Health Assessment and Risk in Ethnic Groups
U.K.: United Kingdom
U.S.: United States
